# Exercise in pregnant women and birth weight: a randomized controlled trial

**DOI:** 10.1186/1471-2393-11-66

**Published:** 2011-09-30

**Authors:** Lene AH Haakstad, Kari Bø

**Affiliations:** 1 Norwegian School of Sport Sciences, Department of Sports Medicine, P.O Box 4014, Ullevål Stadion 0806 Oslo, Norway

## Abstract

**Background:**

Birth weight plays an important role in infant mortality and morbidity, childhood development, and adult health. To date there are contradictory results regarding the role of physical activity on birth weight. In addition, it is questioned whether exercise during second and third trimesters of pregnancy might affect gestational age and increase the risk of preterm delivery. Hence, the purpose of this study was to examine the effect of a supervised exercise-program on birth weight, gestational age at delivery and Apgar-score.

**Methods:**

Sedentary, nulliparous pregnant women (N = 105), mean age 30.7 ± 4.0 years, pre-pregnancy BMI 23.8 ± 4.3 were randomized to either an exercise group (EG, n = 52) or a control group (CG, n = 53). The exercise program consisted of supervised aerobic dance and strength training for 60 minutes, twice per week for a minimum of 12 weeks, with an additional 30 minutes of self-imposed physical activity on the non-supervised week-days.

**Results:**

There was no statistically significant difference between groups in mean birth weight, low birth weight (< 2500 g) or macrosomia (≥ 4000 g). Per protocol analyses showed higher Apgar score (1 min) in the EG compared with the CG (p = 0.02). No difference was seen in length of gestation.

**Conclusion:**

Aerobic-dance exercise was not associated with reduction in birth weight, preterm birth rate or neonatal well-being.

**Trial Registration:**

ClinicalTrials.gov: NCT00617149

## Background

Provided that pregnancy is normal and healthy, the current American College of Obstetrics and Gynecology (ACOG) guidelines promote continuation of pre-pregnancy exercise activities and recommend that sedentary women start exercising during pregnancy [1,2]. According to the present guidelines, all pregnant women are encouraged to be physically active for at least 30 minutes on most days of the week, in the absence of medical or obstetrical contraindications [1,3,4]. Wolfe and Davies [4] recommended that previous sedentary women should start moderate exercise for a minimum of 15 minutes, 3 to 4 times a week and increase to 30 minutes 5 times a week. However, the optimal dose for recreational physical activity during pregnancy remains to be determined, and the impact of prolonged and repeated aerobic exercise on clinical outcomes for mother and infant are still unknown [5,6]. A systematic review associated physically demanding work with increased risk of premature birth [7], whereas a recent large cohort study showed increased risk of early spontaneous abortion with > 7 h/wk of high impact exercise [8]. Potential risk factors of exercise have been listed as fetal hyperthermia with potential teratogenic effects, reduction of oxygenated blood flow (leading to fetal hypoxia) and reduction in essential substrates leading to fetal growth restriction [4].

Birth weight plays an important role in infant mortality and morbidity, childhood development, and adult health [9-12]. Low birth weight babies are at an increased risk for mortality, short term and long term morbidities [13,14]. Another concern is the increasing prevalence of newborns with high birth weight or fetal macrosomia [15,16]. Several studies show that birth weight ≥ 4000 g is associated with acute complications such as prolonged labour, shoulder dystocia, operative delivery and lacerations [17-19]. Long term health risks include diabetes, obesity, metabolic syndrome and some types of cancer [20-22].

Previous studies investigating the effect of exercise during pregnancy and birth weight report inconsistent findings [23-30]. A Cochrane review from 2009, found no effects of maternal exercise on birth weight [31], and concluded that few studies have examined exercise as a determinant of birth weight.

The purpose of the present study was to examine the effect of aerobic dance exercise twice a week, in addition to 30 minutes of moderate self-imposed physical activity on the remaining week-days, on birth weight, including the proportion of small (< 2500 g) and large (≥ 4000 g) newborns in nulliparous previously inactive pregnant women. The research hypothesis of the present study was: Regular attendance to moderate intensity exercise during pregnancy will not result in reduced birth weight in previously inactive women.

## Methods

### Design

This was an assessor blinded RCT, with the primary aim to evaluate the effect of regular exercise on maternal weight gain [31]. The complete study (including this secondary analysis) was conducted in agreement with the most recent CONSORT statement http://www.consort-statement.org.

### Participants

Nulliparous women whose pre-pregnancy exercise levels did not include participation in a structured exercise program (> 60 minutes once per week), including brisk walking (> 120 minutes per week) for the past six months, were eligible for the trial. Other inclusion criteria were ability to read, understand and speak Norwegian, and to be within their first 24 weeks of pregnancy. Exclusion criteria were a history of more than two miscarriages, severe heart disease (including symptoms of angina, myocardial infarction or arrhythmias), persistent bleeding after 12 weeks of gestation, multiple pregnancy, poorly controlled thyroid disease, pregnancy-induced hypertension or pre-eclampsia, diabetes or gestational diabetes, and other diseases that could interfere with participation [32]. In addition, women not able to attend weekly exercise classes were ineligible. Participants were recruited via articles and advertisement in newspapers, health practitioners (physicians, midwives) and websites for pregnant women.

The participants came from the city of Oslo, Norway. In total, 105 women were recruited to the trial from September 2007 to March 2008. All follow-up procedures were completed by November 2008. A priori sample size calculation was only done for the primary outcome (gestational weight gain) of the study. Results in previous studies, have shown that a minimum sample size of 20-50 per group was required to detect a 10% difference in birth weight at the 0.05 level, with a power of 0.80 [25,26,28].

**Figure 1:**
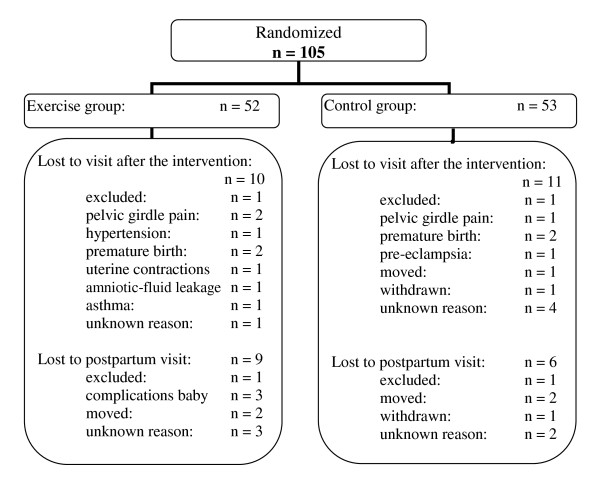
**Trial profile showing the flow of participants through the randomized controlled trial**.

In total, the participants were examined three times during the study period. The first visit was between 12 and 24 weeks of gestation (baseline visit), the second at week 36-38 (after the intervention) and the last 6-12 weeks after delivery (postpartum visit). Each visit lasted approximately 60-75 minutes. Figure 1 illustrates the flow chart, including drop-outs and reasons for withdrawal. Some women who were lost to the second visit and test after the intervention (lost to follow up), re-entered the study at the postpartum examination. There was no financial compensation to the participants.

All participants gave written consent to participate and the procedures followed the World Medical Association Declaration of Helsinki. The project was approved by The National Committee for Medical Research Ethics, Southern Norway, Oslo, Norway (reference number S-05208). The Norwegian Social Sciences Data Services (NNT) provided licence to store and register individual health information (reference number 17804/2/KH).

### Randomization

A secretary, not involved in the assessment or exercise classes, assigned the participants to either an exercise group (EG) or a control group (CG) following a computerised randomization program. A simple randomization procedure was used, and no stratification was done. The principal investigator (LAHH) was not involved in training the women and was blinded to group allocation while assessing the outcome measures, plotting and analyzing the data.

### Intervention

Participants randomized to EG were encouraged to participate in at least two out of three possible one hour aerobic dance classes per week, for a minimum of 12 weeks. Each session started with 5 minutes warm up, followed by 35 minutes of aerobic dance, including cool down. This was followed by 15 minutes of strength training with a special focus on the deep abdominal stabilization muscles (internal oblique and the transverse abdominal muscle), pelvic floor and back muscles. The last 5 minutes included stretching, relaxation and body awareness exercises. The aerobic dance routine included low impact exercises (no jumping or running) and step training. Step length and body rotations were reduced to a minimum, and crossings of legs and sharp and abrupt changes of position were avoided. The exercise-program followed the ACOG exercise prescription [1,2], and all aerobic activities were performed at moderate intensity measured by ratings of perceived exertion at 12-14 (somewhat hard) on the 6-20 Borg's rating scale [33]. The exercise program was choreographed and led by certified aerobic-instructors, and each session included a maximum of 25 participants. Since most participants were working full time, the exercise groups were arranged in the evening.

In addition to joining the scheduled aerobic classes, all women in the EG were asked to include 30 minutes of moderate self-imposed physical activity on the remaining week-days. They were also advised to incorporate short bouts of activity into their daily schedules (e.g. walk instead of drive short distances and to use stairs instead of elevators). Adherence to the exercise classes was controlled by the aerobic-instructors, and the self-imposed daily activity was registered in a personal training diary.

It was not considered unethical to use a control group not receiving treatment in the present study. However, control participants were neither encouraged to, nor discouraged from, exercising, as we considered asking the CG not to exercise to be against current guidelines. In order to treat the two groups identically apart from the experimental intervention, the CG underwent all tests and completed the same interview as the EG.

### Outcome measure

The baseline interview covered demographic information (e.g. age, pregnancy week, smoking habits, education, occupation), assessment of daily life physical activity and sedentary behaviour (at work, transportation and household). The questionnaire has been validated with a portable activity monitor [34]. At the postpartum test, birth weight, length, head circumference, gestational age at time of delivery and Apgar score at 1 and 5 min after birth were registered from labor and delivery records. The main outcome measure was infant birth weight measured in grams. In addition, newborns birth weight was grouped according to low birth weight (LBW) (< 2,500 g), normal birth weight (2,500-3,999 g) and macrosomia (≥ 4,000 g) [35,36]. Secondary outcome measures were gestational age at delivery and Apgar score. Newborn characteristics were obtained from labor and delivery records and interviews with the participants.

### Statistical analysis

The principal analysis was done on an intention to treat basis (ITT). Because, drop-outs rates in the present study were less than 20%, missing values were replaced with the mean value in the EG and CG, respectively [37]. In addition, we performed per protocol analysis based on adherence to ≥ 80% of the recommended exercise sessions (≥ 19 exercise sessions) and compared women with 100% exercise adherence (24 exercise sessions) with the CG [38]. Average infant birth weight was compared between the two groups and the possible difference was tested using a two-sided independent sample t-test. The group differences in proportion of newborns with low birth weight (LBW) (< 2500 g) and macrosomia (≥ 4000 g) were tested by using two-sided *X^2^*-test. Level of statistical significance was set to p < 0.05.

## Results

The participants were predominately of Norwegian descent (n = 94). Countries of origin for the other women were Sweden (n = 5), Uganda (n = 1), Iran (n = 1), Chile (n = 1), Russia (n = 1), Poland (n = 1) and Burundi (n = 1), respectively. Background variables of the 105 nulliparous women randomized to EG (n = 52) or CG (n = 53) are shown in Table 1. There were no statistically significant differences in background variables between the two groups prior to the intervention at mean gestation week 17.7 (SD 4.2).

**Table 1:** Background variables in the exercise and control groups.

Detail	EG (n = 52)		CG (n = 53)	
Age	31.2	(3.7)	30.3	(4.4)
Gestational wk	17.3	(4.1)	18.0	(4.3)
Married/living together	51	(98.1)	52	(98.1)
College/university education	44	(84.6)	45	(84.9)
Sedentary occupations *(> 50% of the working day)*	37	(71.2)	36	(67.9)
Daily smokers *(Do you smoke daily: Yes/No)*	2	(3.8)	1	(1.9)
Height (m)	1.69	(0.1)	1.69	(0.1)
Pre-preg weight (kg)	67.9	(11.4)	68.4	(14.6)
Weight (kg)*	71.8	(11.4)	72.7	(14.3)
Pre-preg BMI (kg/m^2^)	23.8	(3.8)	23.9	(4.7)
Pre-preg BMI≥ 25	13	(25.0)	14	(26.4)

Means with standard deviation (SD) and N (%) (n = 105). No statistically significant differences between groups at baseline

* At baseline test, pregnancy weight was measured using a digital beam scale

In total, 85.7% of the participants met at the postpartum visit where measurements of birth weight was obtained, at mean 7.7 (SD 1.7) weeks postpartum. One woman in the EG and one in the CG were excluded due to twin birth and poorly controlled thyroid disease after the first assessment, respectively. Three drop-outs were due to complications with the baby, five due to relocations and withdrawals, and five were unknown reasons (Figure 1). There was no difference in maternal physical characteristics between the women who completed the study and those lost to follow-up.

Adherence to the EG was mean 17.0 (SD 12.5) sessions and 21 (40.4%) attended ≥ 80% of the exercise sessions. After the intervention period, six of 53 women in the CG reported that they had exercised ≥ 2 times per week for 60 minutes of moderate intensity. None of the exercises performed by the CG were supervised, as opposed to the EG.

### Birth weight

Table 2 summarizes the results of mean newborn birth weight in the EG and CG of the ITT, per protocol analysis and analyzes of women attending 24 exercise sessions. Other newborn characteristics are also presented in Table 2. Excluding the women who reported to exercising regularly in the CG (n = 6) did not change the overall results.

**Table 2:** Newborn birth weight and offspring characteristics in the exercise and control groups (mean (SD) and N (%)), analyzed by intention to treat (ITT), per protocol (≥ 80% of exercise sessions) and analyses of 100% exercise adherence (24 exercise sessions)

	ITT			Per Protocol			100% exercise adherence		
	*EG (n = 52)*	*CG (n = 53)*	*p-value*	*EG (n = 21)*	*CG (n = 53)*	*p-value*	*EG (n = 14)*	*CG (n = 53)*	*p-value*
Birth weight (g)	3477 (424)	3542 (464)	0.4	3451 (450)	3542(464)	0.4	3310 (463)	3542 (464)	0.1
< 2500 (g)	1 (1.9)	1 (1.9)		0 (0)	1 (1.9)		0 (0)	1 (1.9)	
2500 to < 4000 (g)	46 (88.5)	43 (81.1)		18 (85.7)	43 (81.1)		13 (92.9)	43 (81.1)	
≥ 4000 (g)	5 (9.6)	9 (17.0)	0.5	3 (14.3)	9 (17.0)	0.7	1 (7.1)	9 (17.0)	0.6
Birth lenght (cm)	50.2 (2.0)	50.8 (1.9)	0.1	49.8 (1.7)	50.8 (1.9)	0.04	49.5 (1.7)	50.8 (1.9)	0.03
Head circumference (cm)	34.9 (1.4)	35.1 (1.6)	0.5	34.7 (1.4)	35.1 (1.6)	0.3	34.4 (1.7)	35.1 (1.6)	0.2
Gestational age	39.9 (1.2)	39.6 (1.2)	0.2	39.7 (1.0)	39.6 (1.2)	0.8	39.6 (1.1)	39.6 (1.2)	0.9
Apgar score 1 min	8.8 (0.8)	8.6 (1.2)	0.3	9.1 (0.3)	8.6 (1.2)	0.02	9.1 (0.4)	8.6 (1.2)	0.02
Apgar score 5 min	9.6 (0.6)	9.4 (0.8)	0.4	9.6 (0.6)	9.4 (0.8)	0.2	9.7 (0.6)	9.4 (0.8)	0.2
Gender									
- Boys	24 (46.2)	27 (50.9)		11 (52.4)	27 (50.9)		6 (42.9)	27 (50.9)	
- Girls	28 (53.8)	26 (49.1)	0.6	10 (47.6)	26 (49.1)	0.8	8 (57.1)	26 (49.1)	0.9

We did not find statistically significant differences between the two groups in mean birth weight, length, head circumference, and length of gestation, according to ITT-analysis. Per protocol analysis showed a statistical significant difference between the two groups in Apgar score (1 min), with newborns of the EG scoring higher than the CG. No newborn in the EG had a score < 7, compared with two newborns in the CG.

The prevalence of newborns with low birth weight (LBW) (< 2500 g) was 1.9% in both groups. Macrosomia (≥ 4000 g) was 9.6% (5 of 52) and 17% (9 of 53), in the EG and CG, respectively (p = 0.5).

No major adverse effects or health problems resulting from the exercise program were reported. Two preterm deliveries occurred in the EG (gestational age: 36.1 and 36.5) and one preterm delivery in the CG (gestational age: 35.0). There were no reports of miscarriage in either group during this study.

## Discussion

This is one of very few RCTs investigating the effect of a supervised structured exercise program on birth weight. No negative effects of a twice a week 12 week aerobic dance program in 2^nd ^and 3^rd ^trimester of pregnancy in previously sedentary women were found, and there was no statistically significant difference between groups in mean birth weight, low birth weight (< 2500 g) or macrosomia (≥ 4000 g). Regular exercise during pregnancy did not affect gestational age or prematurity.

The strengths of the present study were use of an assessor blinded RCT design, few losses to follow-up and implementation of an exercise program following ACOG recommendations, conducted by certified personnel in a supervised setting. In addition, we aimed at integration of exercises into daily life activities, a focus not reported in other studies [31]. Adherence to the training protocol was registered, and all follow-up procedures were done by the same investigator. A limitation was the adherence to the training program, and that variation in nutritional intake was not assessed. However, EG and CG had similar gestational weight gain [39].

Sample size determination for birth weight was not based on a-priory power calculations. Post priori power calculations showed that we would need 64 subjects in each group to detect a mean difference in newborn birth weight between EG and CG of 230 g (6-7% difference in birth weight), significant at the 5% level with a power of 80%. In addition, post priori calculation of difference in newborns with macrosomia (same alpha and power), showed that 262 participants were needed in each group, respectively. Future studies are warranted and may base the power calculations on the results of the present study.

The results of the present study are difficult to compare with other studies since the prescribed exercise dosages vary widely, in addition to inclusion of different study populations, time in pregnancy and the length of the intervention. Clapp [25] reported that previously physically inactive women who were assigned at gestation week 8 to exercise for 20 minutes 3-5 times per week for the remainder of pregnancy, gave birth to significantly heavier newborns than the control women (3750 g vs. 3490 g, p = 0.05). Hopkins et al [40] reported opposite results and concluded that regular exercise (five sessions of 40 min stationary cycling per week) was associated with lower birth weight (3426 g vs. 3569 g). A recent Cochrane review, involving 258 women and their newborns, concluded that the available data were insufficient to infer important risks or benefits of maternal exercise on birth weight [31]. A meta-analysis based on both experimental, quasi-experimental and cohort studies, concluded that exercise in pregnancy generally does not affect birth weight [41]. Our results support this conclusion.

The clinical importance of a small reduction in mean birth weight is questionable, and it may be more relevant if maternal exercise primarily decreased the number of newborns with macrosomia, which may reduce the risk of prolonged labour, operative deliveries, shoulder dystocia and fetal hypoxia [35]. In the present study, we did not find a significant difference in mean birth weight between EG and CG, nor number of LBW babies. However, we observed that the prevalence of newborns with birth weight ≥ 4000 g was 9.6% (n = 5) in the EG vs. 17% (n = 9) in the CG. This is consistent with findings of Barakat et al [23], showing higher prevalence of macrosomic babies in the control group than in the training group (1.4% vs.10%). In Finland, Kinnunen et al [42], found a 15% incidence of newborns above 4000 g in the control group, whereas there was no newborns exceeding 4000 g in the intervention group. Macrosomic infants have an increased risk of developing diabetes, obesity and metabolic syndrome [35]. Hence, this gives support to start prevention interventions in pregnancy.

It has been discussed that physical activity before pregnancy has a protective effect against macrosomia [35]. In this study we included only previously sedentary women. Other studies have included both exercisers and non-exercisers, and pre-pregnancy physical activity may be a confounding factor linked to birth weight when groups are not comparable at baseline [24;27].

Another interesting finding in the present study was that mean Apgar score of the newborns was higher in the EG compared to the CG at 1-minute. However, by 5-minutes there was no difference. Clinically, the 5-minute score may be more relevant, as this score assesses how well the newborn is adapting to the new environment, compared to how well the baby has tolerated the birthing process (1-minute score). Nevertheless, the results of the present study confirm previous data which showed that moderate intensity aerobic exercise does not negatively affect birth outcomes or gestational age [23,31,43].

The moderate intensity of the exercise classes in the present study, followed the ACOG guidelines [1] and can easily be achieved in most aerobic classes or by brisk walking. However, the present study also demonstrated that it is difficult to motivate former sedentary women to fulfil the ACOG exercise recommendations. A main limitation of the present study is related to the difficulties the participants in the EG had in regularly attending the scheduled aerobics dance sessions. On the other hand, this may represent a realistic picture of the possibilities of recruiting sedentary pregnant women, even in those with low-risk pregnancies. In a recent RCT, the most frequently reported barriers for low adherence to exercise groups were children and household duties, job-imposed limitations, lack of transportation and distance between the woman's home and the fitness club [44]. Few of these factors were present in our study, and why the nulliparous women in the present study did not adhere is difficult to understand. A fitness class of 60 minutes prescribed twice a week, including endurance training of 40 minutes may be considered demanding. Thus, the sedentary women being the target group for this study may have been less motivated to adhere to this specific program. In addition, time management is vital if an exercise program is to be successful.

Unlike most other studies, we did not recruit from one maternity unit, but contacted women across a wide range of sites and settings, varying from newspapers, flyers, maternity clinics and word of mouth. However, RCT's are time consuming and involve cooperation from the participants. Hence, pregnant women who volunteer for such a study may have an interest and be more attentive to these aspects than non-participants, creating a potential risk for selection bias. The pregnant women in this study were healthy nulliparous with a high educational level, and are therefore not representative for all eligible women.

## Conclusions

Aerobic-dance exercise for sedentary pregnant women appeared to be safe and was not associated with any reduction in newborn birth weight, preterm birth rate or neonatal well-being. Further studies on strategies to achieve adherence to exercise protocols among previous sedentary pregnant women are warranted.

## Competing interests

The authors declare that they have no competing interests.

## Authors' contributions

LAHH plotted all data, developed the protocol together with KB, and outlined the manuscript. LAHH is responsible for the data collection and recruited all participants. KB originated the idea for present study, led on its design, and supervised the project. Both authors participated in discussing the design of the study, read and corrected draft versions of the manuscript and approved the final manuscript.

## Pre-publication history

The pre-publication history for this paper can be accessed here:

http://www.biomedcentral.com/1471-2393/11/66/prepub
